# Supplementary material for: Prediction and action in cortical pain processing
Source: Cereb Cortex. 2022 Mar 15;33(3):794–810. doi: 10.1093/cercor/bhac102 (PMC9890457; doi:10.1093/cercor/bhac102)
Supplement: Predict_act_supplementary_revised_220202_bhac102 [file predict_act_supplementary_revised_220202_bhac102.docx]

***Supplementary Materials***

**Prediction and action in cortical pain processing**

Lina Koppel, Giovanni Novembre, Robin Kämpe, Mattias Savallampi, India Morrison

**Pilot studies**

In order to confirm the appropriateness of the task design, we conducted two pilot studies in which participants performed the task outside the scanner. In the original version of the task, the time from Cue offset to S1 onset was 1s and the duration of the press cue was 350 ms (with a 350ms response time window). However, because the effect on response times seemed weaker than predicted in pilot study 1, we conducted an additional pilot study in which we increased the time from Cue offset to S1 onset to 2s in order to allow more time for participants to process the instruction. Because response times were slower than anticipated, indicating a low proportion of trials on which participants succeeded to respond in time to reduce the duration of the upcoming stimulation, we also increased the duration of the press cue (and thus the time window in which participants could affect upcoming pain) to 450ms in order to increase the sense of controllability. The methods and results from each pilot study are described in detail below.

**Pilot study 1**

**Participants.** 28 participants were recruited using the same methods as in the main experiment, except they only needed to meet inclusion criteria for age (at least 18 years old) and not be taking pain relieving medication, antidepressants, anxiolytics, or other medication that may influence the perception of pain. Participants provided written informed consent in accordance with the Declaration of Helsinki and were compensated at 200 SEK/hour. One participant was excluded due to a technical error, leaving 27 participants for analysis. Due to technical issues, twelve participants only completed one of two runs of the task.

**Materials and procedure.** The pain stimuli and task were identical to those reported in the paper, with two exceptions: (1) time from Cue offset to S1 onset was 1s and (2) the duration of the press cue was 350ms. Thus, participants had to respond within 350ms in order to reduce the duration of S2 on controllable trials. Participants were seated at a desk and completed the task on a computer. The experimenter (L.K.) was positioned next to the participant and followed sound cues delivered via headphones (not audible to the participant) indicating the timing of thermode onset and offset for manual stimulus delivery.

**Results.** We performed a 2 × 2 × 2 repeated-measures ANOVA with current stimulation (pain or nonpain), predicted stimulation (pain or nonpain), and action (effctive or ineffective) as within-subjects factors and reaction time as dependent variable. Results are shown in Supplementary Figure 1. There was a significant main effect of predicted stimulation, *F*(1,26) = 5.99, *p* = .021, η_p_^2^ = 0.187, indicating that participants responded faster when the predicted stimulation was painful (*M* = 487 ms, *SE* = 10) than when it was nonpainful (*M* = 502 ms, *SE* = 10). There were no main effects of current stimulation (*p* = .828) or action (although the effect was in the predicted direction, *F*(1,26) = 3.49, *p* = .073, η_p_^2^ = 0.118) and no significant interactions (although the interaction between predicted stimulation and action was significant at α = .10, *F*(1,26) = 3.01, *p* = .094, η_p_^2^ = 0.104).

**
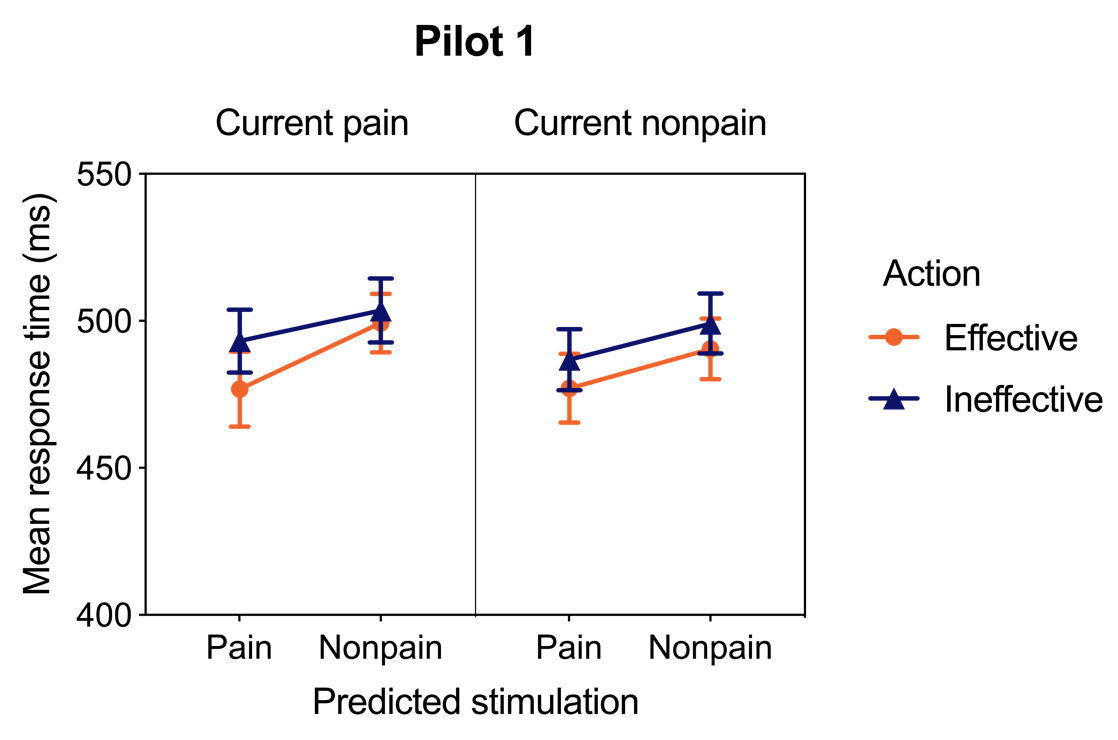
**

**Figure S1.** Mean response time as a function of current stimulation, predicted stimulation, and action in Pilot Study 1. Error bars represent standard errors.

**Pilot study 2**

**Participants.** 21 participants were recruited and compensated as in pilot study 1. Due to technical issues, one participant only completed one of two runs of the task.

**Materials and procedure.** The pain stimuli and task were identical to those reported in the paper. Participants were seated at a desk and completed the task on a computer. The experimenter (L.K.) was positioned next to the participant and followed sound cues delivered via headphones (not audible to the participant) indicating the timing of thermode onset and offset for manual stimulus delivery.

**Results.** We performed a 2 × 2 × 2 repeated-measures ANOVA with current stimulation (pain or nonpain), predicted stimulation (pain or nonpain), and action (effective or ineffective) as within-subjects factors and reaction time as dependent variable. Results are shown in Supplementary Figure 2. There was a significant main effect of action, *F*(1,20) = 7.64, *p* = .012, η_p_^2^ = 0.276, indicating that participants responded faster when the button-press action was effective (*M* = 492 ms, *SE* = 9) than when it was ineffective (*M* = 511ms, *SE* = 9). There were no main effects of current or predicted stimulation and no significant interactions, although there was a close to significant three-way interaction, *F*(1,20) = 4.25, *p* = .053, η_p_^2^ = 0.175. Pairwise comparisons with Bonferroni correction indicated that response times were faster when pain predicted action-effective pain than when (1) pain predicted action-ineffective pain (*p* = .005), (2) pain predicted action-ineffective nonpain (*p* = .042), (3) nonpain predicted action-ineffective pain (*p* = .037), and (4) nonpain predicted action-ineffective nonpain (*p* = .026).


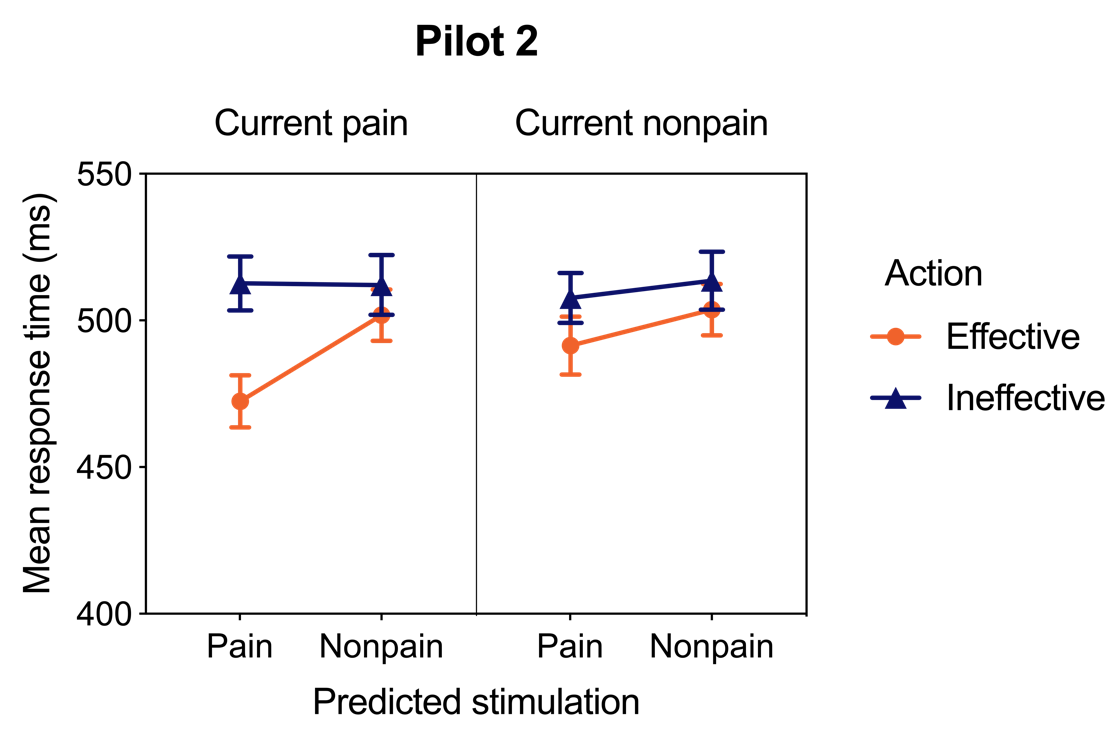


**Figure S2.** Mean response time as a function of current stimulation, predicted stimulation, and action in pilot Study 2. Error bars represent standard errors.


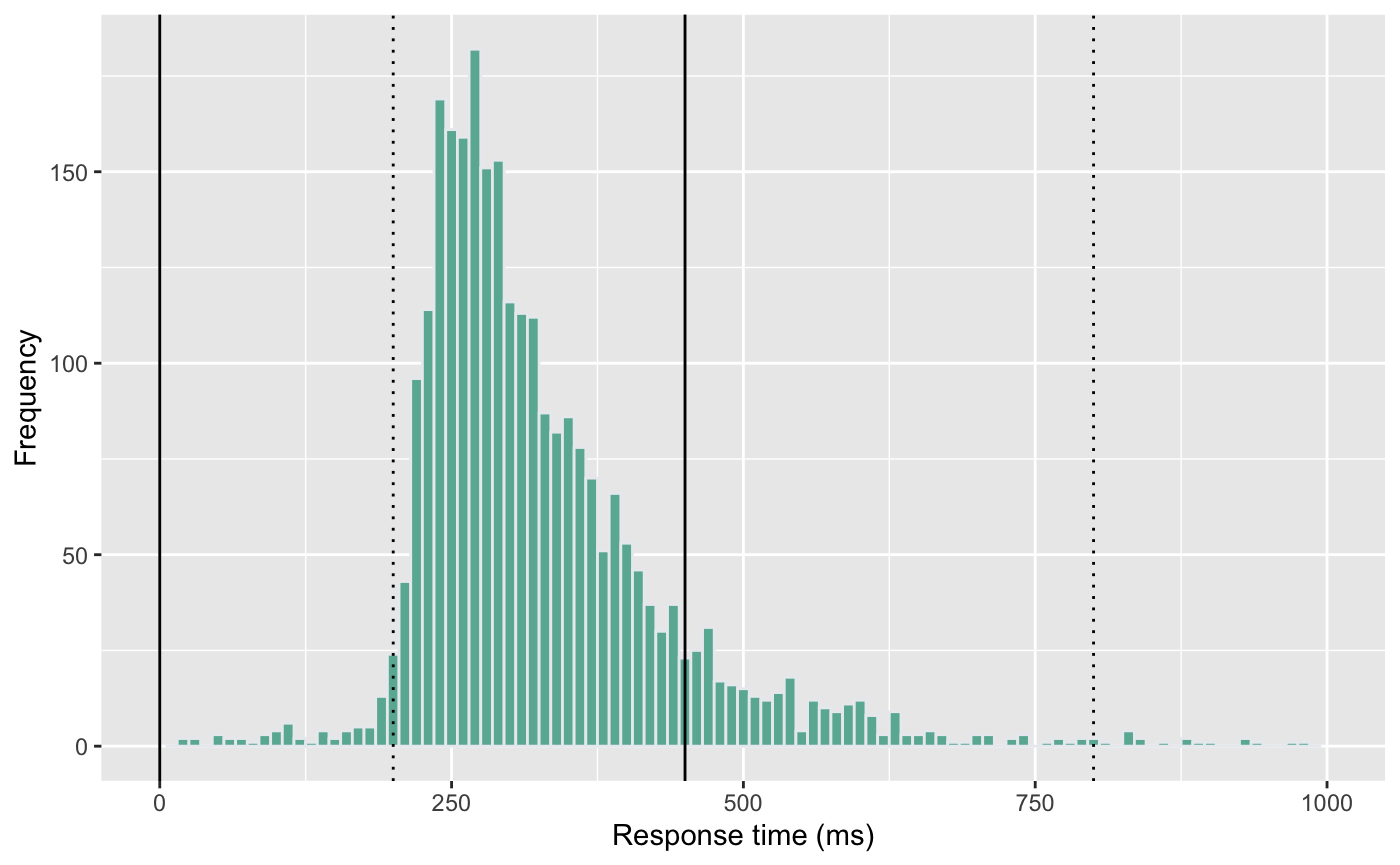


**Figure S3.** Distribution of response times. The solid lines represent the cutoffs for the response window to reduce the duration of upcoming stimulation (0–450 ms) and the dotted lines represent minimum and maximum response times for inclusion in analyses (200 ms and 800 ms, respectively). 85.9% of responses fell within the 450 ms response window. 93.6% of responses fell within the 200–800 ms inclusion cutoffs. We limited the graph at 1000 ms to improve interpretability of the data; as a result, eight observations with rt > 1000 ms are not shown in the figure.


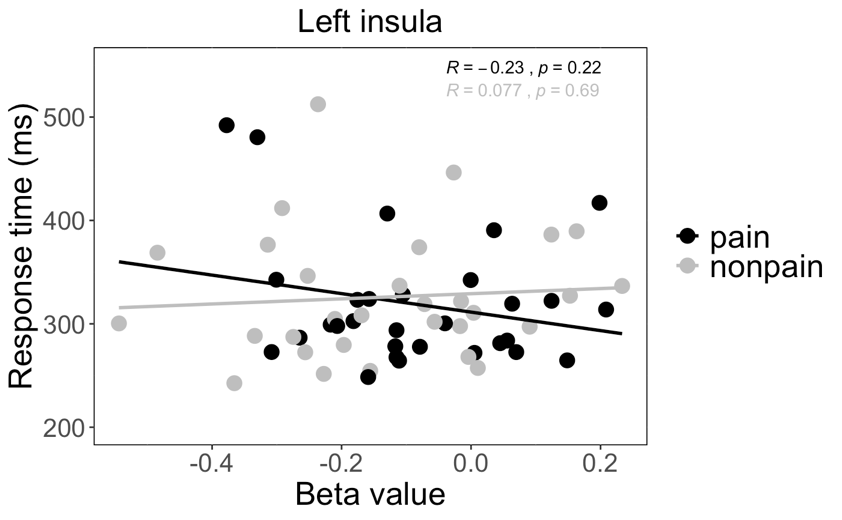

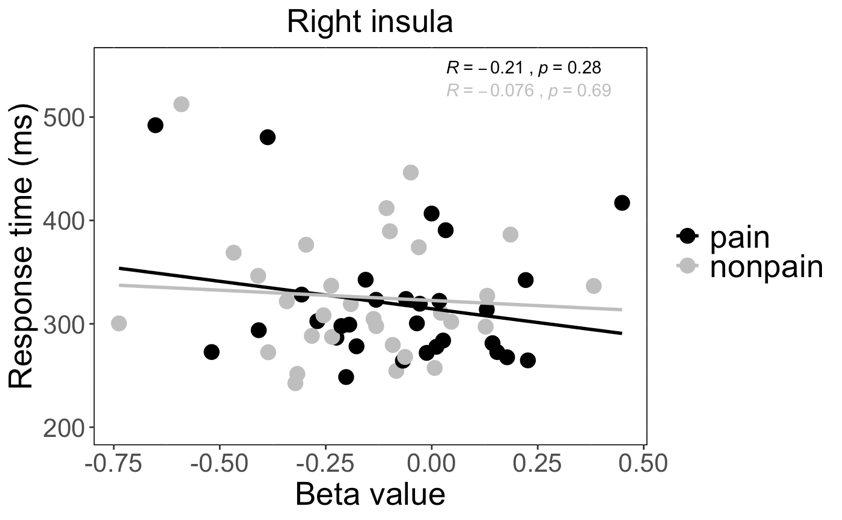


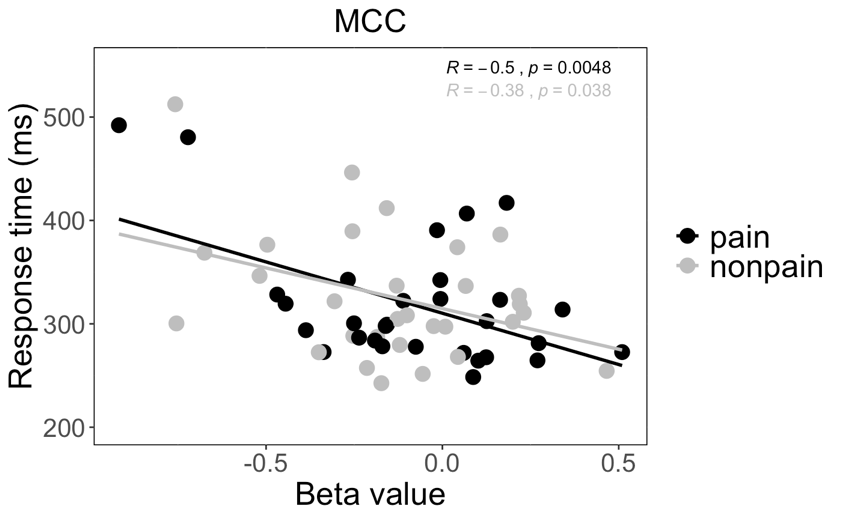


**Figure S4.** Correlations between response times for predicted painful and nonpainful stimuli and ß values of activation in MCC, left insula, and right insula on trials on which current stimulation was painful and action was effective.


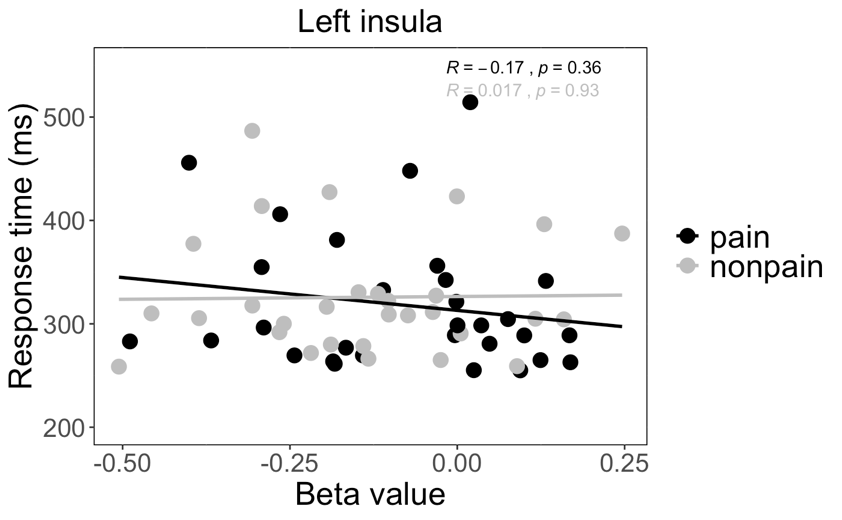

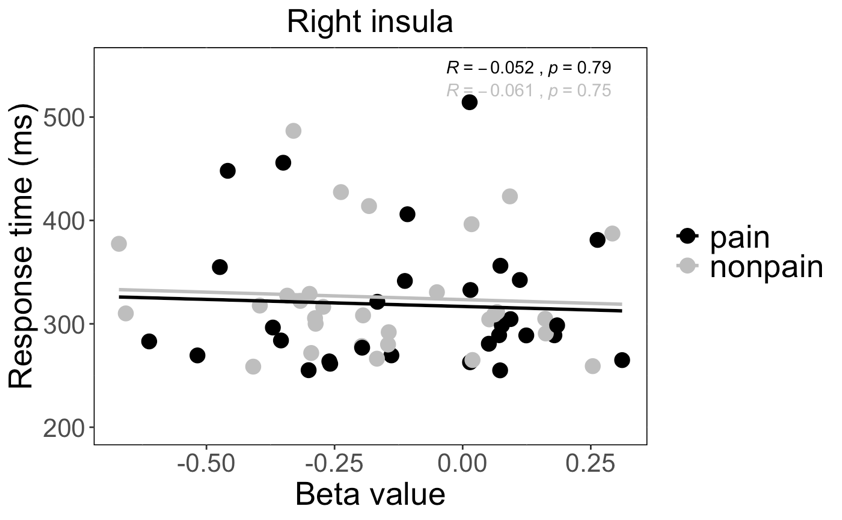


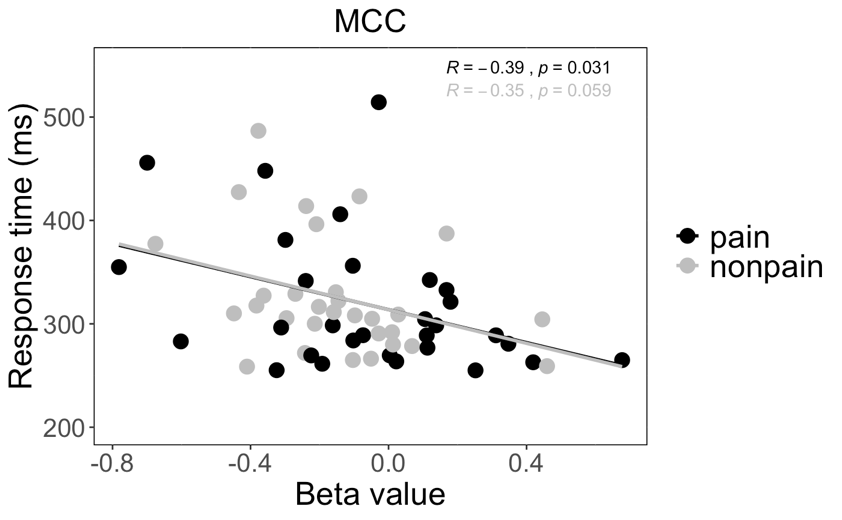


**Figure S5.** Correlations between response times for predicted painful and nonpainful stimuli and ß values of activation in MCC, left insula, and right insula on trials on which current stimulation was nonpainful and action was effective.


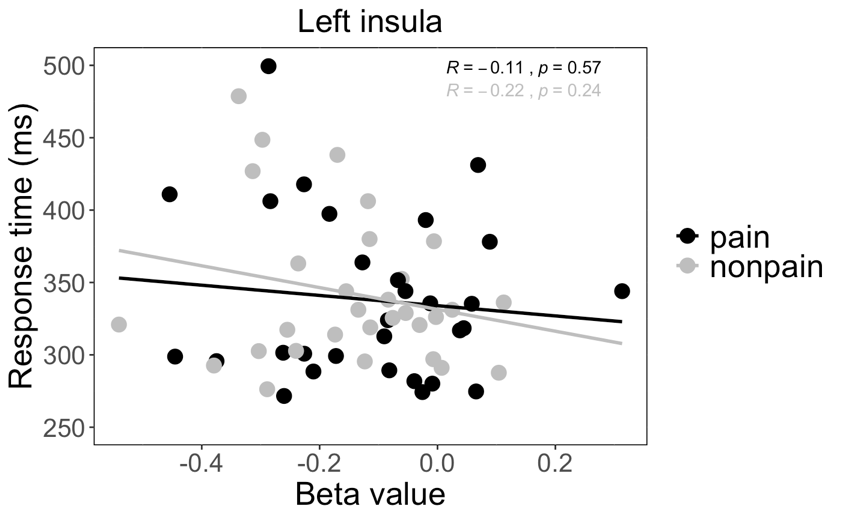

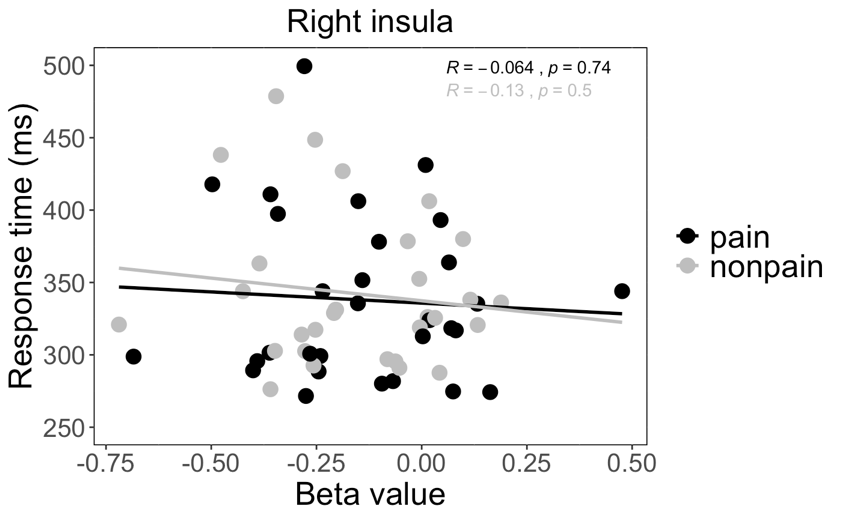


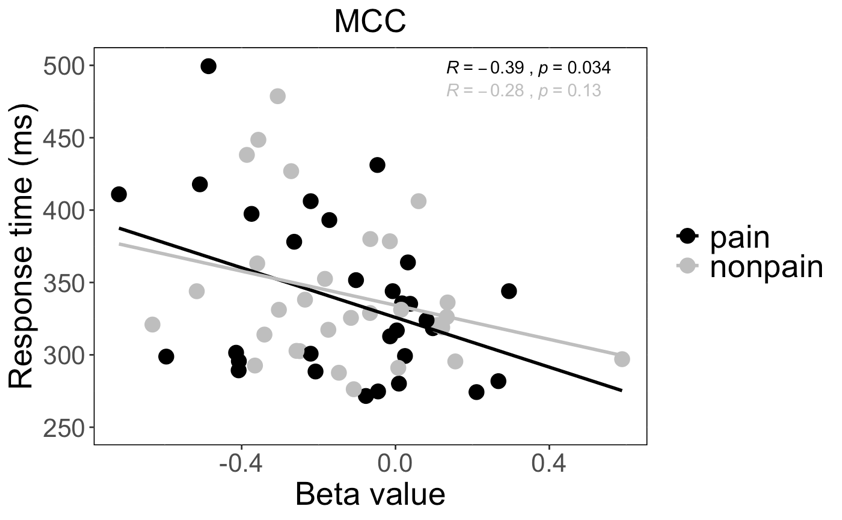


**Figure S6.** Correlations between response times for predicted painful and nonpainful stimuli and ß values of activation in MCC, left insula, and right insula on trials on which action was ineffective.


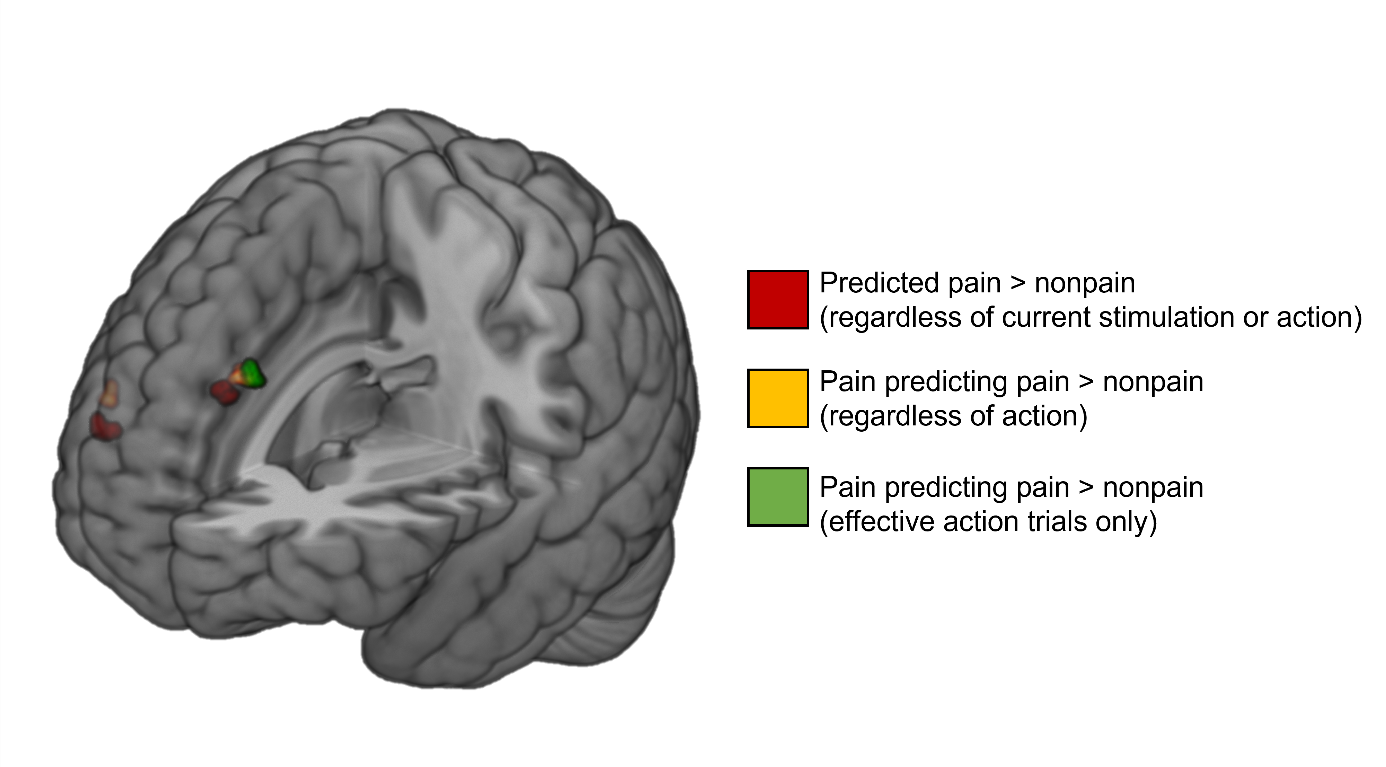


**Figure S7.** Caudorostral organization of predictive and action-related responses in the cingulate. Mapping of unsmoothed group data indicates a selective, partly nested spatial organization within a cingulate subregion likely corresponding to anterior rostral cingulate motor zone (RCZa). A rostralmost cluster (red) showed highest selectivity for predicted pain, regardless of either current stimulation level (painful or nonpainful) or action task level (effective or ineffective). A more caudal, adjacent cluster (yellow) was selective for predicted pain during pain regardless of action task level. The caudalmost cluster (green) was selective for predicted pain during current pain stimulation, in effective action trials only. This spatial organization suggests a shift from less stimulus- or task-bound functional coding of predicted stimuli rostrally, to a greater specificity for meaningful action and current stimulation more caudally.

**Table S1**

ANOVA results for insula and MCC ROIs

|  |  | *F* | *p* | η_p_^2^ |
| --- | --- | --- | --- | --- |
| Right insula | |  |  |  |
|  | Current stimulation | .01 | .907 | <0.001 |
|  | Predicted stimulation | 25.02 | <.001 | 0.463 |
|  | Action | 2.63 | .115 | 0.083 |
|  | Current × predicted stimulation | 3.06 | .091 | 0.095 |
|  | Current stimulation × action | 0.11 | .740 | 0.004 |
|  | Predicted stimulation × action | 2.98 | .095 | 0.093 |
|  | Current × predicted × action | 0.18 | .672 | 0.006 |
| Left insula | |  |  |  |
|  | Current stimulation | 0.06 | .807 | 0.002 |
|  | Predicted stimulation | 17.69 | <.001 | 0.379 |
|  | Action | 1.84 | .185 | 0.060 |
|  | Current × predicted stimulation | 2.30 | .140 | 0.074 |
|  | Current stimulation × action | 0.37 | .545 | 0.013 |
|  | Predicted stimulation × action | 0.31 | .583 | 0.011 |
|  | Current × predicted × action | 5.70 | .024 | 0.164 |
| MCC | |  |  |  |
|  | Current stimulation | 0.01 | .924 | <.001 |
|  | Predicted stimulation | 2.65 | .115 | 0.084 |
|  | Action | 2.18 | .151 | 0.070 |
|  | Current × predicted stimulation | 0.46 | .503 | 0.016 |
|  | Current stimulation × action | 0.41 | .525 | 0.014 |
|  | Predicted stimulation × action | 2.64 | .115 | 0.083 |
|  | Current × predicted × action | 3.42 | .075 | 0.105 |
